# Evolution of C2H2-zinc finger genes and subfamilies in mammals: Species-specific duplication and loss of clusters, genes and effector domains

**DOI:** 10.1186/1471-2148-8-176

**Published:** 2008-06-18

**Authors:** Hamsa D Tadepally, Gertraud Burger, Muriel Aubry

**Affiliations:** 1 Department of Biochemistry, Université de Montreal, C.P. 6128, Succ. Centre-Ville, Montreal, QC, H3C 3J7, Canada

## Abstract

**Background:**

C2H2 zinc finger genes (C2H2-ZNF) constitute the largest class of transcription factors in humans and one of the largest gene families in mammals. Often arranged in clusters in the genome, these genes are thought to have undergone a massive expansion in vertebrates, primarily by tandem duplication. However, this view is based on limited datasets restricted to a single chromosome or a specific subset of genes belonging to the large KRAB domain-containing C2H2-ZNF subfamily.

**Results:**

Here, we present the first comprehensive study of the evolution of the C2H2-ZNF family in mammals. We assembled the complete repertoire of human C2H2-ZNF genes (718 in total), about 70% of which are organized into 81 clusters across all chromosomes. Based on an analysis of their N-terminal effector domains, we identified two new C2H2-ZNF subfamilies encoding genes with a SET or a HOMEO domain. We searched for the syntenic counterparts of the human clusters in other mammals for which complete gene data are available: chimpanzee, mouse, rat and dog. Cross-species comparisons show a large variation in the numbers of C2H2-ZNF genes within homologous mammalian clusters, suggesting differential patterns of evolution. Phylogenetic analysis of selected clusters reveals that the disparity in C2H2-ZNF gene repertoires across mammals not only originates from differential gene duplication but also from gene loss. Further, we discovered variations among orthologs in the number of zinc finger motifs and association of the effector domains, the latter often undergoing sequence degeneration. Combined with phylogenetic studies, physical maps and an analysis of the exon-intron organization of genes from the SCAN and KRAB domains-containing subfamilies, this result suggests that the SCAN subfamily emerged first, followed by the SCAN-KRAB and finally by the KRAB subfamily.

**Conclusion:**

Our results are in agreement with the "birth and death hypothesis" for the evolution of C2H2-ZNF genes, but also show that this hypothesis alone cannot explain the considerable evolutionary variation within the subfamilies of these genes in mammals. We, therefore, propose a new model involving the interdependent evolution of C2H2-ZNF gene subfamilies.

## Background

The sequencing of the human genome uncovered that a large number of gene families are often arranged in a clustered organization [1-3]. C2H2 zinc finger (C2H2-ZNF) genes make up ~2% of all the human genes and represent the second largest gene family in humans after the odorant receptor family [4-7]. The first identified members of the C2H2-ZNF family were *Xenopus *TFIIIA and *Drosophila *Kruppel and thus genes of this family are often called zinc finger genes of the TFIIIA or Kruppel type [6,8].

Most of the characterized C2H2-ZNF genes code for transcription factors which bind DNA through their zinc finger region; others bind RNA and their exact function is as yet unknown [9,10]. The zinc finger region is composed of a basic structural unit of 28 amino acids (CX_2–4_CX_3_FX_5_LX_2_HX_3–4_HTGEKPYX, where X is any amino acid), called the zinc finger motif that is often repeated in tandem. The two cysteines and two histidines in this motif interact with a zinc ion, stabilizing the proper folding of this motif [11-13]. C2H2-ZNF proteins often contain an effector domain that is always located N-terminal to the zinc finger region, such as the KRAB (Kruppel-Associated-Box), SCAN (SRE-ZBP, CTfin51, AW-1 and Number18 cDNA) and BTB (Broad-Complex, Tramtrack and Bric-a-bric) domains. The first two domains are vertebrate-specific [14-16], while BTB is also present in insects. The KRAB domain includes the KRAB A box (~38 amino acids) involved in transcriptional repression, and often a second box, usually KRAB B (~32 amino acids) or in a few cases KRAB b or KRAB C (~21 amino acids) box [17-20]. The KRAB A box and the second KRAB B, b or C boxes are encoded by separate exons often prone to alternative splicing. The SCAN, also called the leucine-rich (LeR) domain (~84 amino acids) [21], mediates protein-protein interactions through dimerization [22,23]. The BTB domain (~120 amino acids) is a dimerization domain that also acts as a repression domain in some cases [24]. In contrast to the SCAN and KRAB domains, which are only present in C2H2-ZNF proteins, the BTB domain is also found as a part of actin-binding proteins [16]. C2H2-ZNF proteins are grouped into different subfamilies based on the type of N-terminal effector domain present.

Initial studies on the C2H2-ZNF gene family focused on human chromosome 19, which is particularly enriched in clusters of these genes [25,26]. More recent studies dealt more specifically with the KRAB subfamily [18,27-29]. The current view is that C2H2-ZNF genes have undergone a massive expansion during vertebrate evolution, primarily by tandem duplication [18,27,29-32]. Yet, this view may be biased, because it is extrapolated from subsets of C2H2-ZNF genes.

In this report, we reconstruct a global picture of the evolution of the C2H2-ZNF gene repertoires during mammalian speciation that is based on a comprehensive catalogue of all human C2H2-ZNF genes and their syntenic counterparts present in clusters in other mammals. Our study clearly demonstrates that this gene family expanded and contracted not only in human but across mammals, and in a lineage-specific fashion. In addition, we discovered evolutionary change of individual C2H2-ZNF orthologs involving both differential duplication of zinc finger motifs and loss of N-terminal effector domains. This led us to propose a model for the interlinked evolution of SCAN, SCAN-KRAB and KRAB subfamilies, underlining the importance of comparing complete gene repertoires rather than specific subfamilies for gaining insights into the possible orthologous relationships between genes from various genomes. Our data indicates that during speciation of mammals, divergent evolutionary trends at the level of individual C2H2-ZNF genes as well as the entire family characterized the evolution of the C2H2-ZNF genes.

## Results

### Compilation of a comprehensive catalogue of human C2H2-ZNF genes

**Figure 1:**
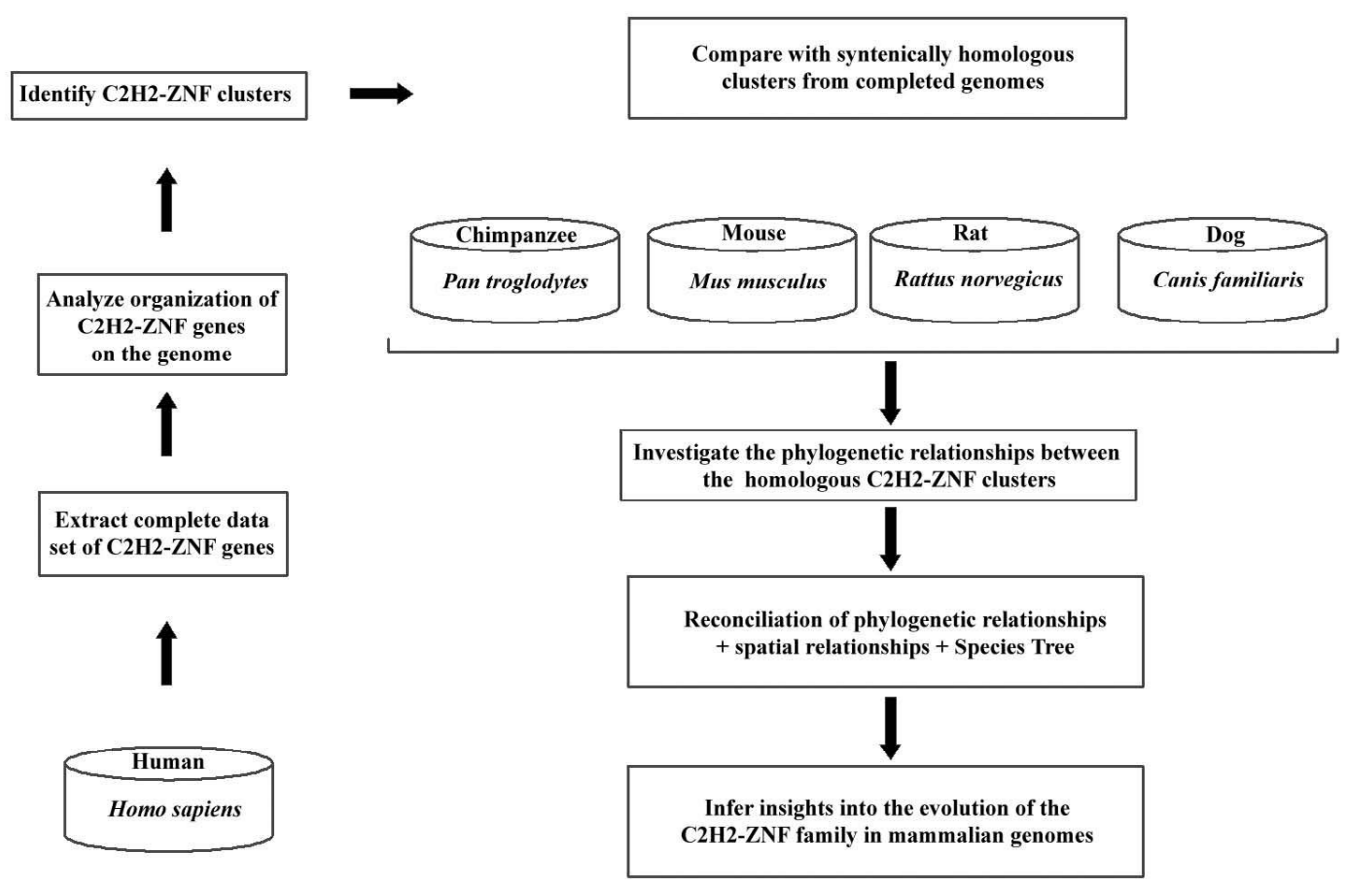
Flowchart overview of the approach used in the study.

Previous studies reported the existence of at least 564 C2H2-ZNF genes in the human genome and suggested that this family may include approximately 700–800 genes [7,14]. As a first step to study the evolution of C2H2-ZNF genes, we established a comprehensive catalogue of the C2H2-ZNF genes in the human genome. By conducting an extensive similarity search (see Methods and Figure 1 for a flowchart of the study) we identified 718 C2H2-ZNF genes (see Additional File 1). Of the 718 genes, 66 are annotated as pseudogenes in GenBank. For all genes, we determined their exact position on the chromosomes, their orientation, the number of finger motifs and the effector domains.

**Figure 2:**
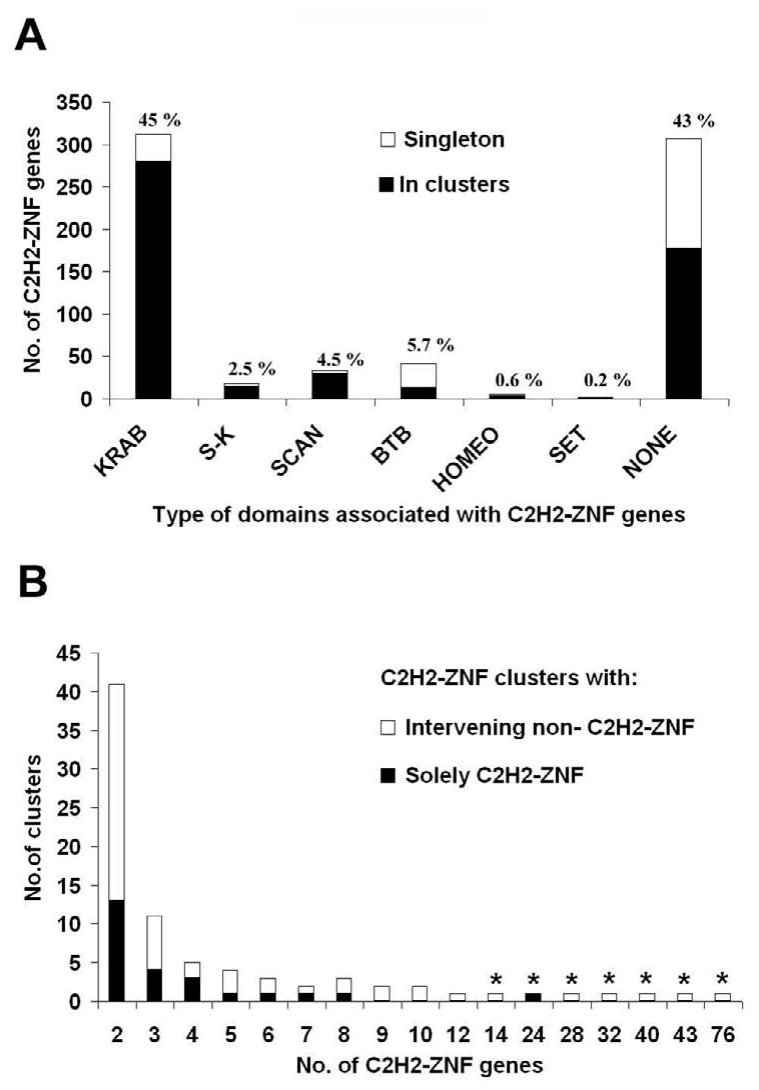
**Distribution of all the singletons and clustered genes from the various human C2H2-ZNF sub-families and gene composition of the C2H2-ZNF clusters**. **A) **The number of genes belonging to the various C2H2-ZNF subfamilies are shown as well as the proportion of genes found as singletons or as part of clusters. C2H2-ZNF genes associated with KRAB and SCAN domains are more often found to be clustered. S-K = C2H2-ZNF containing both a SCAN and a KRAB domain. NONE = C2H2-ZNF without any conserved domain associated. The percentage distribution is mentioned on top of each bar for each sub-family. **B) **The number of C2H2-ZNF clusters is shown with respect to the number of genes present in each cluster. The proportion of clusters composed solely of C2H2-ZNF without any intervening gene or with intervening genes other than C2H2-ZNF (Non-C2H2-ZNF) is also represented. An asterisk identifies large clusters present on human chromosome 19.

C2H2-ZNF genes are distributed across all chromosomes of the human genome (see Additional File 2). As reported earlier, chromosome 19 has the highest number [2] and density of these genes, i.e., 40% (289 of the 718), whereas this chromosome corresponds to only 2.1% of the human genome. More than half (58%) of the C2H2-ZNF genes encode conserved N-terminal domains, the KRAB, SCAN and BTB domains (see Figure 2A), typically involved in transcriptional regulation [16,33] and form different C2H2-ZNF subfamilies. Further, we discovered two additional domains typical of transcription regulators, the SET and HOMEO domains that are also encoded by C2H2-ZNF genes. While the KRAB subfamily represents almost half of the C2H2-ZNF genes (45%), SET and HOMEO C2H2-ZNF genes together with members of all the other subfamilies account for only a small percentage (~12%) of the C2H2-ZNF genes (see Figure 2A).

### Clustered organization of human C2H2-ZNF genes

It was reported earlier that human chromosome 19 is particularly rich in tandemly duplicated C2H2-ZNF genes and that KRAB C2H2-ZNF genes are also found in clusters on several other chromosomes [32,34]. In order to trace the duplication history of the entire C2H2-ZNF repertoire, we studied the distribution of these genes across the whole human genome. Two consecutive C2H2-ZNF genes were considered to belong to a cluster if the distance between them is ≤ 500 Kbp, regardless of the presence of other genes or pseudogenes within the cluster (see Methods). Using this definition, we identified 81 human C2H2-ZNF clusters accounting for 72% of the total number of C2H2-ZNF genes (518 of the 718) (see Additional Files 2 and 3). The remaining genes are dispersed as singletons. Among these clusters, 31% include exclusively tandemly organized C2H2-ZNF genes with no other intervening genes (see Figure 2B and Additional File 3). The number of C2H2-ZNF genes per cluster ranges from 2 to 76 with an average of 6. As illustrated in the Figure 2B, about 75% of the total numbers of C2H2-ZNF clusters have between two to six genes. Consistent with previous reports, chromosome 19 not only has the largest number of C2H2-ZNF clusters (see Additional File 2) but also hosts the largest clusters (>12 genes) (see Figure 2B and Additional File 3).

We find that the large majority of KRAB (89%) and SCAN (90%) types of C2H2-ZNF genes are arranged in clusters (see Figure 2A and Additional File 2). This contrasts with the BTB subfamily of C2H2-ZNF genes or those lacking effector domains, which occur more often as singletons in the genome. An analysis of the composition of individual clusters revealed that two-third of the clusters contains a mixture of various C2H2-ZNF subfamilies (see 'mixed clusters', Additional File 3). The few clusters made up of a single C2H2-ZNF gene subfamily ('pure clusters') are of small size (< 4 genes).

### Identification and comparison of syntenic C2H2-ZNF clusters across mammals

With the ultimate goal to study the evolution of zinc finger genes, we identified and compiled clusters in completely sequenced mammalian genomes (i.e., chimpanzee, mouse, rat and dog) that are syntenically homologous to those of human. Syntenically homologous clusters were identified by the genes flanking each cluster. All the C2H2-ZNF genes found within the delimited syntenic regions were then identified using a TBLASTN search (see Methods). The 81 human C2H2-ZNF clusters and their syntenic counterparts in other mammals are listed in Additional File 4, which also includes information on the orientation of the genes in the clusters, their associated domains, the number of zinc finger motifs and the flanking genes.

**Figure 3:**
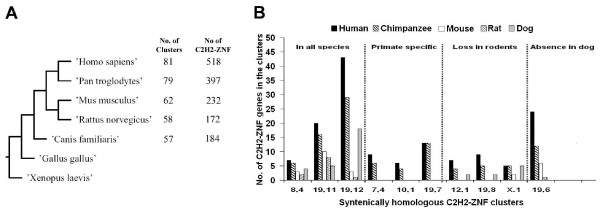
**Differential expansion and loss of C2H2-ZNF clusters in five mammalian genomes**. **A) **Evolution of the C2H2-ZNF repertoires in primates, rodents and dog. The number of C2H2-ZNF clusters and the total number of C2H2-ZNF genes found in these clusters are mentioned on the species tree. Since *Xenopus laevis *and *Gallus gallus *C2H2-ZNF genes are used as an outgroup in phylogenetic studies, these species are also positioned on the tree. **B) **A graphical representation of different scenarios seen in the evolution of human C2H2-ZNF clusters and their syntenically homologous C2H2-ZNF clusters in chimpanzee, mouse, rat and dog. The human clusters selected and named on the graph as well as their syntenic counterparts were 1) present in all species, 2) primate-specific, 3) lost in rodents or 4) absent in dog. For each human C2H2-ZNF cluster named on the graph and described in Additional File 3, the first number indicates the chromosome number and the second is the number attributed to that cluster on the chromosome. Additional File 5 provides a more comprehensive graphical representation including the 40 human clusters that contain at least 3 C2H2-ZNF genes and their syntenic counterparts in the four other mammals.

Primates (*Homo sapiens *and *Pan troglodytes*) stand out for their large number of both C2H2-ZNF clusters and genes within them, as compared to rodents (*Mus musculus *and *Rattus norvegicus*) and dog (*Canis familiaris*) (see Figure 3A). The most parsimonious explanation is that a large expansion of C2H2-ZNF genes occurred in primates, and more particularly in human (518 genes in human *versus *397 in chimpanzee) after divergence from rodents and canines. Rat has slightly fewer C2H2-ZNF genes than dog (7%), but 25% less than mouse. Considering the evolutionary relationship of the species (see Figure 3A), these data suggest not only species-specific duplication events, as reported earlier [29,31,32], but also loss of family members (suggested here in rodents) during the evolution of mammals. Differential species-specific expansion was reported previously for a subset of genes from the human ZNF45 subfamily on chromosome 19 compared with its mouse counterpart [29]. Furthermore, expansion of the human KRAB C2H2-ZNF subfamily was also shown earlier based on draft versions of the genomes of chimpanzee, mouse and dog [27]. However, evidence of C2H2-ZNF gene or cluster loss could not be obtained in these previous studies as it required detailed analysis of more than two completely sequenced genomes.

### Comparing individual syntenic clusters in the mammalian genomes

To distinguish whether differences in the number of C2H2-ZNF clusters are due to species-specific gene gain or loss, we systematically compared individual syntenic clusters in the five mammalian genomes studied. The results of this analysis point to a differential evolutionary history in mammals. About 60% of the human clusters (49) have syntenically homologous counterparts in all the species studied, indicating that these C2H2-ZNF clusters predate the divergence of dog, rodents and primates (see Additional File 4 and Additional File 5). In addition, we found (i) primate specific clusters (14, including 2 human specific clusters), (ii) clusters, present in primates and dog, that were lost in rodents (8 clusters including 3 present in mouse but absent in rat) and (iii) clusters present in primates and rodents but absent in dog (10 clusters) (examples in Figure 3B). Essentially all the primate clusters have larger number of genes than rodent or dog clusters, which reflect a global primate-specific expansion of C2H2-ZNF genes (see Additional File 5). Further, in 40% of all primate clusters, those from human contain more C2H2-ZNF genes than those from chimpanzee. This indicates that most of the evolutionary changes (duplication and/or loss) occurred late in the primate branch. A similar pattern was seen in rodents, where almost all mouse C2H2-ZNF clusters exhibit more genes than their syntenic rat clusters. While these results illustrate that the C2H2-ZNF gene family is rapidly and independently evolving within different lineages, insights into the role of gene duplication and loss in the history of this gene family required rigorous phylogenetic analysis as described below.

### Phylogeny of C2H2-ZNF clusters in mammalian genomes

**Figure 4:**
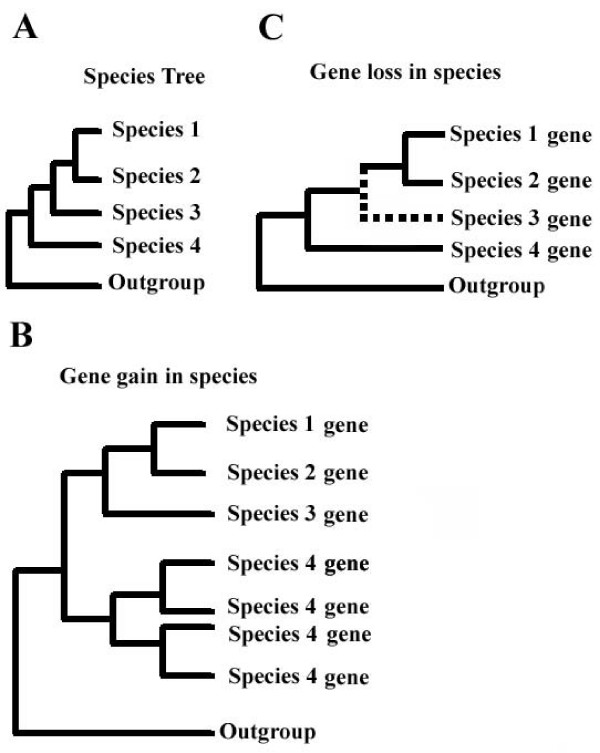
**Evolutionary scenarios in phylogenetic trees**. The different kinds of evolutionary scenarios seen in phylogenetic trees are shown. **A) **Species tree showing the evolutionary relationship between the species, 1, 2, 3 and 4. **B) **A species-specific gain of genes appears as a clade including a single homolog from one species and multiple homologs from the other. Phylogeny between genes from species 1, 2, 3 and 4, respectively is shown. Gene gain in species 4 is observed. **C) **Species-specific gene loss appears as the absence of a corresponding ortholog for one species on the tree and is deduced from the evolutionary relationships of the species considered with the other species. Gene loss occurred in species 3.

**Figure 5:**
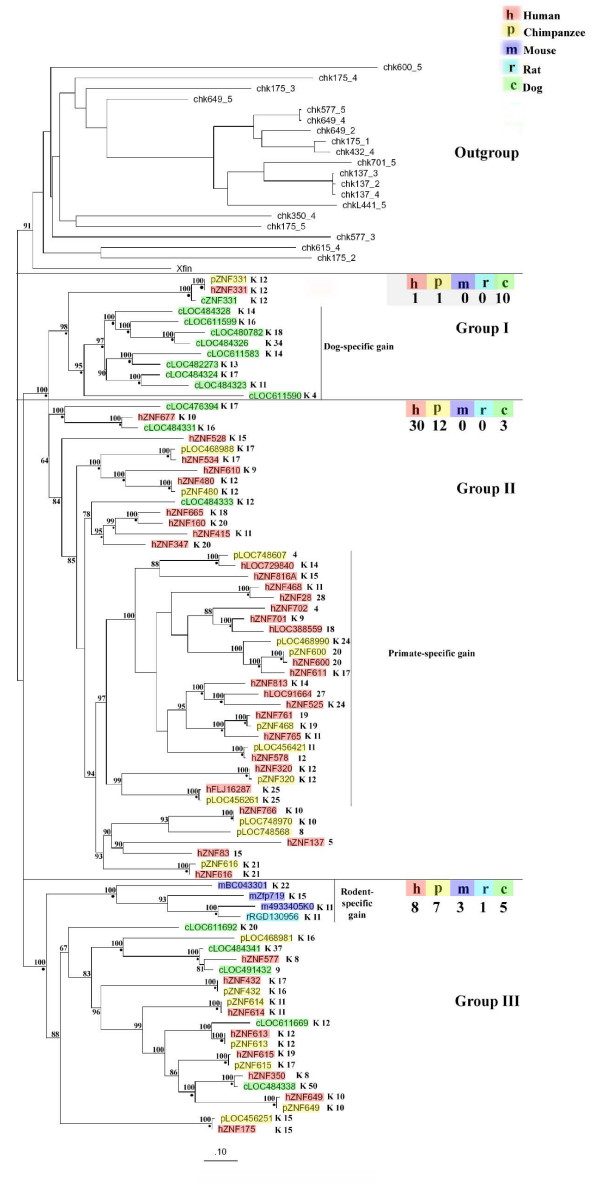
**Phylogenetic analysis of C2H2-ZNF genes in human cluster 19.12 and its syntenic counterparts in other mammals**. A phylogenetic tree was built using the amino acid sequences corresponding to the zinc finger regions of the various human C2H2-ZNF genes from cluster 19.12 and their syntenic counterparts in chimpanzee, mouse, rat and dog. The tree was generated using a maximum likelihood method (RaxML) and verified using a bayesian method (MrBayes). 346 sites from 101 sequences (including the 20 outgroup sequences from chicken and *Xenopus*) were used in the analysis. The tree is divided into three major Groups (I-III). A tabulation of the number of genes present in each group is indicated for each species (h: human, p: chimpanzee, m: mouse, r: rat, c: dog). The bootstraps values are indicated for each node on the tree. A small black circle is also represented at each node in cases where the posterior probability value is equal to 1.00. This cluster contains only C2H2-ZNF genes that are either from the KRAB subfamily or that do not encode any conserved N-terminal domain. Next to the name of each C2H2-ZNF gene, the presence of an N-terminal KRAB domain is indicated by a K and number of zinc finger motifs is mentioned. A clear evidence of differential expansion is seen in primates and dog. Loss of C2H2-ZNF in the rodent lineage is also observed.

To address the relative contribution of gene duplication and loss in the evolution of C2H2-ZNF genes in mammals, we focused our study on selected large human C2H2-ZNF clusters and their syntenic counterparts in four other mammals. We expected that larger clusters would be more informative and possibly more representative of the whole genomes. Because of the clear evolutionary scenarios observed in the tree, we present here a detailed phylogenetic analysis of the second largest human C2H2-ZNF cluster (43 genes) located on chromosome 19q13.4, that we named cluster *19.12*, and of its syntenic clusters (see Additional Files 3 and 4) in other species. For phylogenetic analysis, we used the predicted amino acid sequences of the zinc finger regions. Genes annotated as pseudogenes in Genbank were not considered in the phylogenetic analysis. Our total data set of C2H2-ZNF sequences from the human cluster *19.12 *and their syntenic homologs in chimpanzee, mouse, rat and dog consists of 101 protein sequences, including the outgroup sequences from *Xenopus *and chicken. We constructed a phylogenetic tree using Maximum Likelihood and Bayesian methods. We subdivided the tree into three groups (see Figure 5) based on the kind of evolutionary scenarios observed i.e., one-to-one and one-to-many orthologous relationships between genes as well as gene loss as defined in Figure 4. The number of C2H2-ZNF sequences from each species is highlighted for each group. Two of these groups are monophyletic with significant (≥ 95%) support in both the Maximum Likelihood and Bayesian analysis (Group I and III).

A detailed analysis of the tree revealed four clades that underwent species-specific expansion, and two clades, with gene loss in some species. For example, a dog-specific expansion is seen in the monophyletic Group I, which includes three clustering genes from human (hZNF331), chimpanzee (pZNF331) and dog (cZNF331), which in turn grouped within a larger clade containing nine additional C2H2-ZNF genes from dog. In addition, this clade indicates a loss in rodents, due to the absence of any mouse or rat genes. Group I alone illustrates how both species-specific duplication in dog and loss in rodents can account for the higher number of genes seen in dog C2H2-ZNF clusters as compared to rodents.

Group II shows more pronounced expansion in human as seen in several clusters. For example, one of the primate-specific clades includes 17 human genes and 7 chimpanzee C2H2-ZNF genes (see Figure 5). Of the 17 human genes present in the clade, only 6 genes show a one-to-one orthologous pairing with chimpanzee genes. Another well supported clade (100% bootstrap) includes a single human gene (hZNF677) clustered with two dog genes (LOC484331 AND LOC476394). In this clade, the absence of a chimpanzee or rodent counterparts to these three genes suggests a loss in these species. For chimpanzee, however, loss by pseudogenization is possibly involved (see physical maps described below); note that the percentage of C2H2-ZNF genes annotated as pseudogenes was higher in chimpanzee than in human C2H2-ZNF clusters (see Additional File 4: '62 chimpanzee vs. 25 human pseudogenes').

In Group III, the relationship of the four rodent genes with the dog and primate genes could not be resolved (bootstrap < 95%). Still, a rodent-specific clade revealed a duplication exclusively in mouse (to the exclusion of rat), and also exhibited a higher number of genes in mouse, as seen in several other cases in our study.

### Superimposition of the phylogenetic trees on the physical maps of clusters

**Figure 6:**
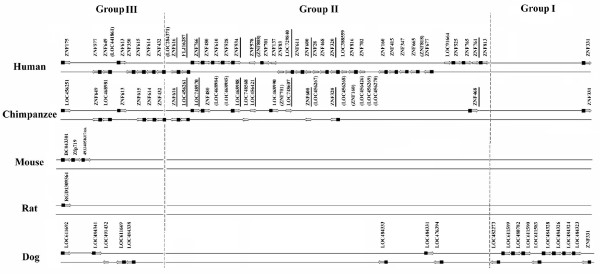
**Physical maps showing the organization of the human C2H2-ZNF genes from cluster 19.12 localized on 19q13.4 and its syntenically homologous counterparts in other mammals**. For the large C2H2-ZNF cluster 19.12 and its syntenically homologous counterparts in chimpanzee, mouse, rat and dog, each C2H2-ZNF gene is represented by an open arrow which indicates its orientation on the chromosome strands (this excludes the pseudogenes whose names appear in parenthesis). The presence of a conserved N-terminal KRAB domain is indicated by a square positioned in front of the open arrow representing the gene. Genes identified as orthologs, based on the phylogenetic tree and physical maps, are underlined and are aligned vertically on their respective chromosomes. Dotted lines separate the genes belonging to Group I, Group II and Group III defined in the phylogenetic tree (Figure 5). The two species-specific groups from dog and primates are seen in Group I and Group II, respectively.

Comparison of the species tree, gene trees and physical map information of cluster 19.12 and its syntenic counterparts provide better insights into the processes underlying the evolution of the C2H2-ZNF clusters in the mammalian species studied. The phylogenetic tree obtained for cluster 19.12 (see Figure 5) suggests a simultaneous differential expansion and loss of C2H2-ZNF genes throughout evolution. In perfect agreement with the phylogenetic tree, genes of the monophyletic groups I and III were found to be physically clustered together on the chromosomes across mammals (see Figure 6). Evidence for a tandem duplication event is provided by the comparison of the relationship within C2H2-ZNF genes of Group I on the tree with their spatial relationships in the physical maps, showing that the sequences of the dog clade form a tandem array on the chromosome (see Figure 5). In addition to tandem duplication of individual genes within this group, e.g., cLOC484324 and cLOC484323 (see Figure 5) which are next to each other on the chromosome and exhibit the same orientation (see Figure 6), we also discovered tandem duplication of multiple genes. For instance, three genes (LOC482273, LOC611599, LOC480782; orientation -, +, +) appear as a tandem repeat of three other genes (LOC611583, LOC484328, LOC484326; orientation -, +, +) in this group (see Figures 5 and 6).

Group II mainly contains primate-specific C2H2-ZNF genes that cluster on the phylogenetic tree in two well supported clades (≥ 97% bootstrap), plus a sub-group with weaker support (93% bootstrap). Almost all these genes also cluster physically together on the chromosome. Human orthology assignments for ten of the twelve chimpanzee genes from Group II (underlined in Figure 6) were corroborated by two lines of evidence i.e. from the phylogeny, and from the topology on the chromosome. Noticeably, only 7 out of 10 of the C2H2-ZNF ortholog pairs from this primate-specific cluster exhibit the same number of zinc finger motifs and the same type of N-terminal domain, suggesting that gain or loss of zinc finger motifs and effector domains often occurs within orthologs.

### Species-specific variation in the number of finger motifs and the presence of N-terminal conserved domains

**Figure 7:**
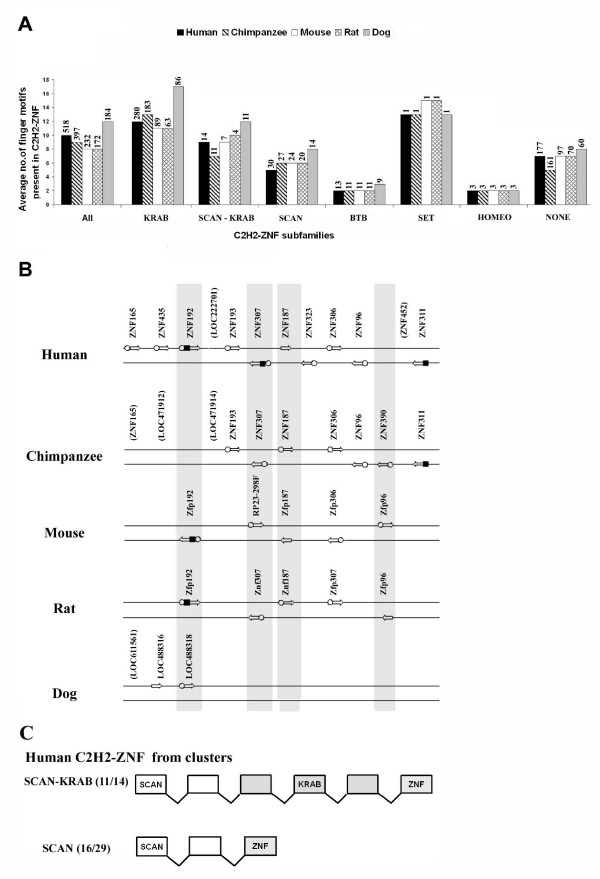
**Variation in the numbers of zinc finger motifs in mammals and in the presence of conserved N-terminal domains in orthologs**. **A) **The average number of zinc finger motifs was calculated for all the C2H2-ZNF genes from the 81 human clusters identified and their corresponding syntenically homologous clusters in the other mammals. For each species, the average number of zinc finger motifs for the total C2H2-ZNF genes (All) and for members of the various C2H2-ZNF sub-families is presented. For each category, the number of genes in each species is listed above the bars in the following order (human, chimpanzee, mouse, rat and dog). **B) **For the human C2H2-ZNF cluster 6.2 (chromosome 6p22.1) and its syntenically homologous counterparts in chimpanzee, mouse, rat and dog, each C2H2-ZNF genes is represented by an open arrow which indicates its orientation on the chromosome strands; this excludes the pseudogenes whose names appear in parenthesis. For these clusters which contain C2H2-ZNF genes that are from the KRAB or SCAN subfamily or that do not encode any conserved N-terminal domain, the presence of a conserved N-terminal is indicated by as square for a KRAB domain or an open circle for a SCAN domain both being positioned in front of the open arrow representing the gene. Genes identified as orthologs, based on the phylogenetic tree and physical maps, are aligned vertically on their respective chromosomes. Cases where orthologs from the different mammals do not consistently share the same effector domain (s) are marked by a grey box. C) Exon-Intron organization of most human C2H2-ZNF genes from the SCAN-KRAB and SCAN subfamilies. 80% of SCAN-KRAB (11/14) and 55% of the SCAN (16/29) C2H2-ZNF genes found in clusters in human have the presented exon-intron structures shown. The exons encoding the SCAN, KRAB (A box) and ZNF are indicated.

When analysing the C2H2-ZNF genes from the 81 human clusters and their syntenic homologs in mammals, we noticed that the average number of zinc finger motifs varied among the C2H2-ZNF gene subfamilies. Noticeably, in all the mammalian species studied, genes with KRAB and SCAN-KRAB motifs have a higher number of zinc finger motifs than those from the other subfamilies (see Figure 7A). For example, members of the KRAB subfamily have an average of 10 to 17 zinc finger motifs, while members of the BTB subfamily have only 2 to 3 (see Figure 7A). We also noted species-specific variation in the number of zinc finger motifs within mammalian C2H2-ZNF genes. In particular, dog tends to have a much higher number of zinc finger motifs in most C2H2-ZNF gene sub-families (see Figure 7). Strikingly, LOC484264, a KRAB C2H2-ZNF gene from dog exhibits 70 zinc finger motifs, which is to our knowledge the highest number reported. Study of cluster 19.12 (see Figure 5) illustrates more specifically the trend of dog genes to possess more zinc finger motifs; the dog LOC484338 (Group III), for example, has six times more zinc finger motifs than its human ortholog. Furthermore, the dog LOC484326 has nearly twice as many motifs as its closest paralog LOC480782 (Group I) (see Figure 5). This indicates a quite recent and drastic expansion of zinc finger motifs within dog C2H2-ZNF genes, after the separation of dog from rodents and primates. In several cases, the C2H2-ZNF mammalian orthologs revealed differences in their numbers of finger motifs even within primate (as mentioned before) or within rodent lineages (see finger motif numbers in Additional File 4 for genes presented in Figure 7B).

**Figure 8:**
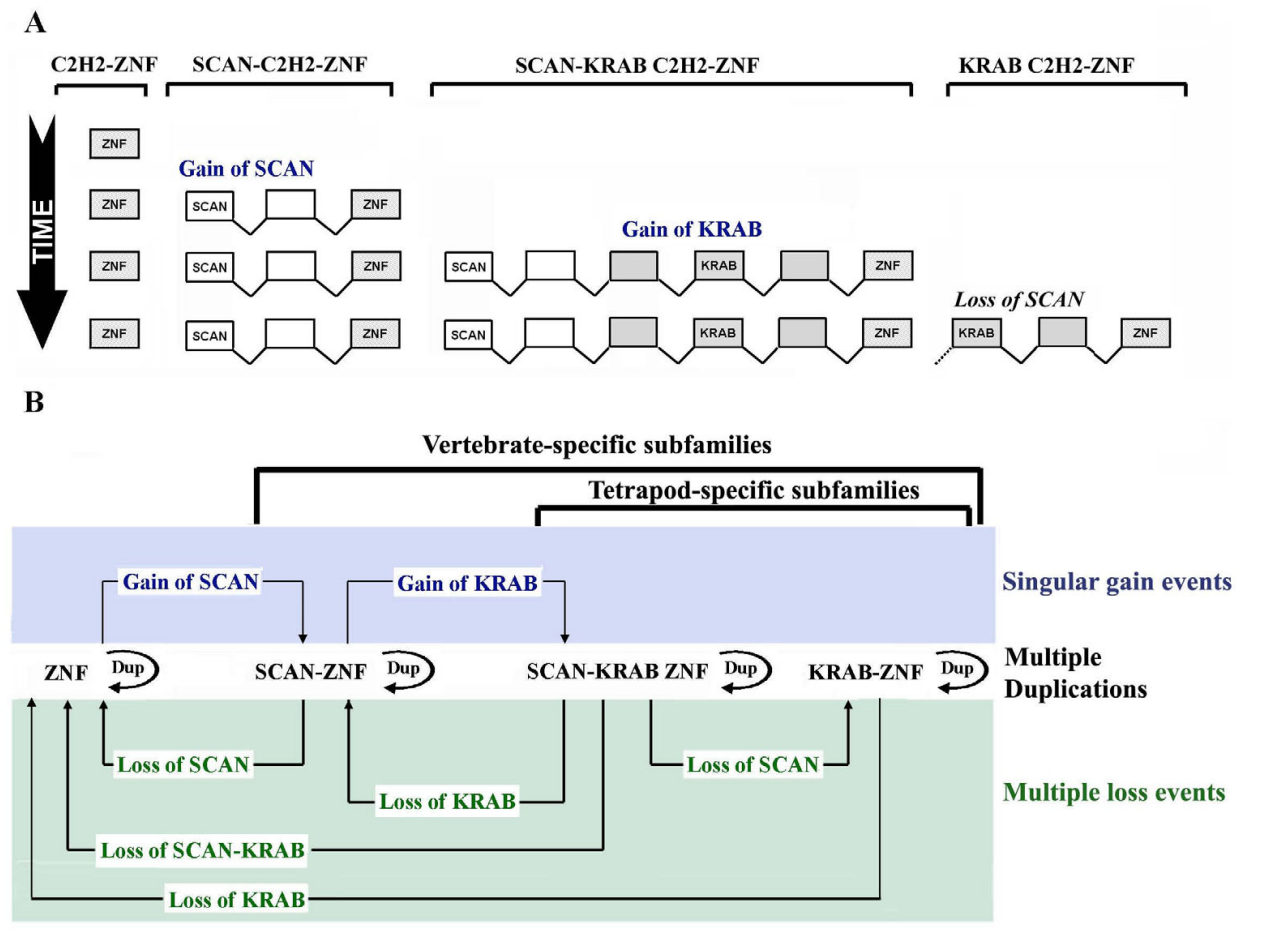
**Model for the evolution of the SCAN, SCAN-KRAB and KRAB C2H2-ZNF subfamilies**. **A) **Sequential events leading to the birth of SCAN and SCAN-KRAB and KRAB C2H2-ZNF subfamilies. Most of the SCAN, SCAN-KRAB and KRAB C2H2-ZNF genes have the exon-intron structure shown (boxes represent exons). Birth of new families may have occurred presumably by an exon shuffling mechanism leading first to the acquisition of a SCAN domain by a C2H2-ZNF gene and later of a KRAB domain by a SCAN C2H2-ZNF gene. In most SCAN-KRAB C2H2-ZNF genes, a single exon is found in between the exon encoding the KRAB A box (identified as KRAB) and the exon encoding the zinc finger domain (ZNF). This exon encodes in most instances the so-called KRAB B, b, or C boxes. KRAB C2H2-ZNF subfamily emergence involved the loss of the SCAN domain from SCAN-KRAB gene (s). **B) **Dynamic evolution of C2H2-ZNF genes after birth of the SCAN and SCAN-KRAB subfamilies through gene duplication and recurrent loss of effector domains. A first SCAN C2H2-ZNF gene appeared in an ancestor of vertebrates following the gain of a SCAN domain by a C2H2-ZNF gene; duplication then led to the establishment of the SCAN C2H2-ZNF subfamily. The gain of a KRAB domain at the emergence of tetrapods by a SCAN C2H2-ZNF gene gave rise to a SCAN-KRAB C2H2-ZNF gene. This was followed by duplication and establishment of the SCAN-KRAB subfamily. Loss of the SCAN domain by deletion or sequence degeneration from some SCAN-KRAB C2H2-ZNF genes followed in many instances by duplication of the resulting KRAB C2H2-ZNF genes led to the expansion of the KRAB C2H2-ZNF subfamily. Loss of SCAN or KRAB domains by deletion or degeneration from SCAN, SCAN-KRAB and KRAB C2H2-ZNF subfamilies is seen as a recurrent theme shaping the repertoires of the C2H2-ZNF subfamilies.

In addition to the difference in the number of finger motifs in C2H2-ZNF orthologs and paralogs, we also found a variation in the presence of the N-terminal effector domains. As an example, orthologs of the C2H2-ZNF genes in the human cluster 6.2 show a variation in the presence of the KRAB or SCAN domains (see Figure 7B), suggesting frequent and multiple losses and/or gains of KRAB and SCAN domains during evolution. To reconstruct these events, we analyzed in detail the exon-intron structure and sequences of these genes (see Methods). Serendipitously, this analysis led us to the observation that a large majority of the C2H2-ZNF genes belonging to the SCAN-KRAB or SCAN subfamilies had each a typical exon-intron organization (see Figure 7C). The human SCAN-KRAB ZNF192 and the chimpanzee SCAN ZNF187 and their respective orthologs in other species presented in Figure 7B physical map exhibit these predominant exon-intron structures. Among the orthologs of the human SCAN-KRAB ZNF192, the dog LOC488318 (see Figure 7B) seems to be an exception as it encodes only a SCAN domain in spite of having a SCAN-KRAB exon-intron structure. However, by comparing the sequences of the third exon after the SCAN domain in dog with those of human, mouse and rat which each encodes a KRAB domain, we discovered significant nucleotide sequence similarity (>82%) that was unrecognizable at the deduced protein sequence level. This indicates that the loss of the KRAB domain in dog was due to sequence degeneration. Similarly, while the chimpanzee ZNF187 and its rat ortholog encode a SCAN domain, a degenerate SCAN domain was identified in the corresponding exon of their human and mouse orthologs. For the human SCAN-KRAB ZNF307, we noticed that it exhibits an exon-intron organization typical of SCAN-KRAB C2H2-ZNF genes (see Figure 7C), whereas its orthologs in the chimpanzee, mouse and rat encode solely a SCAN domain and present an exon-intron structure more typical of SCAN C2H2-ZNF gene. However, it was found that in chimpanzee, a sequence similar to the KRAB sequence (99% at the nucleotide level) was embedded in the intron preceding the exon encoding the zinc finger domain, but no KRAB-related sequence could be detected in the rodent orthologs. Thus, either the KRAB sequence was gained in the primate lineage or lost in the rodent lineage. For reasons explained in the discussion (Model proposed in Figure 8), we believe that loss is the more likely hypothesis.

## Discussion

Comparative studies in genome research have focused on the extensive similarities existing between the human genome and the genomes from various other model organisms, which provide valuable insights into the biological function and the aetiology of human diseases. However, differences among genomes have received less attention, in spite of the importance they may have in the physiological, morphological and behavioural traits that distinguish species. A few studies on various gene families, such as the odorant receptor family, pointed to some differences existing between genes of closely related species [35-39]. Our comprehensive study of the C2H2-ZNF gene family reveals that there is an extensive variation of the C2H2-ZNF gene content and organization, as well as in the domain composition of orthologous genes among mammals. We also provide the first clear demonstration of the contribution of gene loss in the C2H2-ZNF family during evolution, which occurs at the level of clusters, genes and their functional domains. We also provide the first genome-scale confirmation of the rapid evolution of C2H2-ZNF gene clusters that occurs independently within related mammalian species, which also supports conclusions drawn from smaller-scale studies on individual genes, clusters and C2H2-ZNF subfamilies [27,29,30,32]. Altogether our data led us to propose a new model for the interdependent evolution of the main C2H2-ZNF subfamilies (see Figure 8B) as discussed below. Although it is possible that the release of improved genome assemblies of non-human sequences may lead to the detection of a few new C2H2-ZNF genes or of incorrectly assembled C2H2-ZNF loci, these are unlikely to change any conclusions drawn from this study.

### Substantial variation in family size and clustering of C2H2-ZNF genes across mammals

We report here the first complete catalogue of all human C2H2-ZNF gene clusters and their syntenic homologs in chimpanzee, mouse, rat and dog. This catalogue reveals that in human, a large proportion of the genes from the C2H2-ZNF family (>70%) are organized in clusters. Comparative studies of the five mammalian genomes indicated that the total number of genes found in clusters varied considerably, from 172 in rat to 518 in human. Significantly, human and mouse have a larger number of clustered C2H2-ZNF genes (>30%) as compared to chimpanzee and rat, respectively, indicating that independent evolutionary events occurred after the divergence of the two primates (within the last ~6–10 million years) and two rodents (within ~30–46 million years). We distinguish two kinds of events: first, a variation in the number of C2H2-ZNF genes in syntenically homologous clusters and second, the existence of lineage- and species-specific clusters in primates, rodents and canines. This can be accounted for by independent evolution of C2H2-ZNF genes in these closely related species. Previous studies focusing on KRAB C2H2-ZNF genes from human chromosome 19 had identified and analyzed a primate-specific cluster [26] including members of the primate-specific ZNF91 subfamily of C2H2-ZNF [30]. Other studies on the KRAB C2H2-ZNF subfamily also identified a differential expansion between a human KRAB C2H2-ZNF cluster and its syntenic counterpart in mouse [29], and more recently other species-specific expansions have been found, from analyses based on draft sequences of various mammalian genomes [27]. Here, we illustrate and confirm at a larger scale the existence of an on-going process of genome dynamics including several lineage- and species-specific rearrangements and a continuous repertoire expansion taking place independently in all evolutionary branches, particularly in primates. This finding was only possible through the analysis of a complete catalogue of all the subfamilies of C2H2-ZNF clusters and their syntenic counterparts in five mammalian species.

### Gene duplication and loss: two counteracting forces in the evolution of C2H2-ZNF genes

An overview of the 81 human C2H2-ZNF clusters identified here revealed that a third of them are pure clusters (containing 2 to 24 C2H2-ZNF genes), i.e., they are not interspersed with other genes. Earlier observations of pure C2H2-ZNF gene clusters have led to the hypothesis that C2H2-ZNF genes in primates have expanded massively by tandem duplication [25-27,40-42]. We revisited this question based on our catalogue of human C2H2-ZNF clusters and their syntenic counterparts in chimpanzee, mouse, rat and dog. Based on a reconciliation of both the superimposition of gene trees onto the known species trees [43] and the physical maps, we confirm gene duplication and loss. Our results clearly show that both gene gain and gene loss events have occurred multiple times and independently in all the mammals studied. Combined with physical map data, our phylogenetic studies indicate that the expansion of C2H2-ZNF genes that occurred during the evolution of the five species studied, results from the combined action of single-gene duplication and multiple gene duplication (for instances, duplication of all or part of the genes within a cluster). These duplication events were, however, counteracted by the loss of individual genes or clusters as exemplified in several cases where related genes or clusters found in primates and canids were absent in one or both of the two rodents studied. Our study represents the first clear demonstration of the involvement of gene and cluster loss in the evolution of C2H2-ZNF genes and suggests that during mammalian evolution the duplication events outnumbered the loss events. Furthermore our results provide convincing support to the idea that the C2H2-ZNF gene family evolved according to a "Birth and Death" model as proposed by Nei and colleagues [44,45]. According to this model, new genes are created by duplications, including tandem duplication and block (multigene) duplication (birth). While certain copies remain relatively unchanged in the genome for a long time, others diverge functionally by acquiring a new function. Some are deleted from the genome or become pseudogenes following deleterious mutations (death through elimination or inactivation). In the case of C2H2-ZNF genes, pseudogenization seems to be limited as suggested by expression studies and statistical analysis, which showed positive selection based on the analysis of specific clusters [27,40]. This sets the C2H2-ZNF family apart from other gene families such as the olfactory receptor gene family [36,46]. Noticeably, gene loss by pseudogenization is prominent in the olfactory receptor family, with humans accumulating a higher number of pseudogenes as compared to other primates and mouse [37-39]. These variations in the numbers of pseudogenes and functional genes have been associated with the differential chemosensory dependence in these species [47,48]. In comparison, beyond their role as transcription regulators, only a few C2H2-ZNF proteins have known functions [49]. Further studies of C2H2-ZNF genes in mammals are needed to shed light on the functional consequences of different repertoires of these genes in different species. Until now, the clustered organization of these genes has made knock-out studies in animal models inefficient, likely due to redundancy. However, based on a better knowledge of the organization/content of C2H2-ZNF genes in the various genomes, large chromosomal deletions of pure C2H2-ZNF clusters or other types of gene disruption or targeting approaches could provide insights into the functions of these genes in various animals.

### Evolution of C2H2-ZNF genes through gain and loss of finger motifs and N-terminal effector domains

Evidence of the variation in the numbers of zinc finger motifs among orthologs was previously reported for a subset of C2H2-ZNF genes on human chromosome 19 and their mouse homologs [18]. It was shown that this variation is due to both differential duplication of finger motifs and loss by degeneration. In our study, such variation in the number of zinc finger motifs among orthologs was observed repeatedly among all five mammals. Since the zinc finger motifs are flexible, with the ability to bind DNA, RNA and/or proteins, changes in the sequences of zinc finger motifs and their number could differentially alter binding specificities and thus protein function. Changes in both the number of C2H2-ZNF genes and in the number of finger motifs in orthologous genes may be important determinants of species-specific functions.

The rapid evolution of the C2H2-ZNF genes observed in the mammalian lineage was not limited to the variation in the number of genes and zinc finger motifs. Variation was also observed in the presence of N-terminal effector domains, such as SCAN or KRAB, which could be accounted for by either gain or loss of these motifs in the various species. Loss by sequence degeneration of both the SCAN and the KRAB sequences was confirmed in several cases in our study. In certain cases, loss or gain could not be distinguished. A puzzling question that remains, however, is whether gain of KRAB and SCAN sequences occurred multiple times within C2H2-ZNF genes. It is indeed difficult to explain why these effector domains are always found in association with and N-terminal to the zinc finger motifs of C2H2-ZNF proteins, and that the SCAN domain is always positioned N-terminally to the KRAB domain of SCAN-KRAB C2H2-ZNF proteins. Interestingly, by analyzing the exon-intron structure of C2H2-ZNF genes from all clusters, we found that most SCAN and SCAN-KRAB C2H2-ZNF genes have each a typical exon-intron structure (see Figure 7C and Figure 8A). This suggests that the acquisition of SCAN and KRAB sequences by C2H2-ZNF genes are most likely singular events. This led us to propose a new model described in Figure 8. Considering that the SCAN domain is present in all vertebrates and is more ancient than the KRAB domain which is only present in tetrapods, we suggest that first, a SCAN-C2H2 ZNF gene was formed in an ancestor of vertebrates through the gain of SCAN sequence and that later, after the emergence of the tetrapods, the gain of a KRAB sequence gave rise to a SCAN-KRAB C2H2-ZNF gene (Figure 8B). These two gain events possibly occurred through an exon-shuffling mechanism. Diversification of the C2H2-ZNF repertoires from each subfamily then occurred through on-going gene duplications, and loss by deletion or degeneration of the SCAN and/or KRAB sequences. As implied by this model, the birth of the SCAN-KRAB C2H2-ZNF subfamily occurred earlier than that of the KRAB C2H2-ZNF subfamily. This is consistent with previous data showing that SCAN-KRAB-ZNF genes do not group together onto one evolutionary clade but intermix with KRAB-ZNF genes in phylogenetic trees of the KRAB sequence [18,27,27]. On the whole, our model is in agreement with the fact that C2H2-ZNF orthologs often belong to different C2H2-ZNF subfamilies as defined by domains and with our observation of intermingling within many C2H2-ZNF clusters of C2H2-ZNF genes from the SCAN, KRAB and SCAN-KRAB subfamilies. Our study clearly indicates that the evolution of C2H2-ZNF subfamilies is tightly linked and stresses that the assignment of proper orthology requires comprehensive analysis of all C2H2-ZNF genes rather than the individual analysis of specific C2H2-ZNF subfamilies. It also points to the importance of loss/contraction, whose role in the dynamics of evolution is often underestimated. The underlying mechanisms in the expansion of C2H2-ZNF genes and the functional consequences of variation in their repertoires across mammals are yet unclear. It may be that these variations contribute to changes in the control of gene expression/chromatin compaction leading to species-specific morphogenetic programs or cognitive functions in animals.

## Methods

### Collection of human C2H2 zinc finger genes

We conducted an extensive similarity search to identify the complete repertoire of C2H2-ZNF genes in the human genome (assembly NCBI 36). First, we identified all the genes annotated as C2H2 and/or Kruppel zinc finger genes by performing an initial text term search via Entrez [50]. Second, we used PROSITE [51] to identify all the proteins which had a zinc finger motif of the C2H2 type as well as the N-terminal effector domain, if present.

From these searches, the genomic coordinates, chromosome number, position on the chromosome, number of fingers and identified domains were collected for each of the gene and protein sequences (initial dataset). A TBLASTN [52] search (e-value cutoff 1e-3) was done against the genome using each of the gene sequences from the initial dataset as a query. The blast hits were used to generate the final dataset of all the identified C2H2-ZNF genes (see Additional File 1).

### Identification of C2H2-ZNF gene clusters in the human genome

We analyzed the relative positions of C2H2-ZNF genes in the human genome in order to identify the C2H2-ZNF clusters. A distribution of the distances between neighboring C2H2-ZNF genes in the human genome is presented in Additional File 6. Two consecutive C2H2-ZNF genes are said to belong to a cluster if the distance between them is ≤ 500 Kbp regardless of the presence of other genes within the cluster, a threshold classically used in gene family studies [36]. Clusters were determined for each human chromosome. For the cluster name, the first number corresponds to the chromosome number on which the cluster is found and the second to the number attributed to the cluster.

### Identification of mammalian C2H2-ZNF clusters syntenically homologous to human clusters

We searched for clusters homologous to the human C2H2-ZNF clusters (i.e. syntenically homologous clusters) in other mammals for which complete genome sequences are available. The assemblies used for *Pan troglodytes*, *Mus musculus*, *Rattus norvegicus *and *Canis familiaris *were chimpanzee Pan Tro- 2.1, mouse NCBI m36, rat RGSC 3.4 and dog Can Fam 2.0. We used the linkage maps of Ensembl [53]; assignment of syntenic clusters is based on the genes flanking each human cluster and which were mapped in all the species. Four flanking genes at each extremity were mapped in most instances. Then, we conducted TBLASTN analysis of the syntenic regions comprised between the flanking genes, using as queries the amino acid sequence of the zinc finger region from all the human C2H2-ZNF genes from the corresponding region identified in this study. A hit with e-value ≤ 1e-4 confirmed the respective homologous clusters in the five mammalian genomes. A comprehensive catalogue of the human C2H2-ZNF clusters and their syntenic counterparts in other mammals is compiled in Additional File 4.

### Phylogenetic analysis

Phylogenetic analysis was conducted using the amino acid sequences of the zinc finger region (identified using PROSITE) of C2H2-ZNF genes from selected human clusters and their syntenically homologous clusters in chimpanzee, mouse, rat and dog. Genes containing less than three zinc finger motifs were not considered in the phylogenetic analysis (noise is expected to be too high if sequences of only 56 amino acids corresponding to 2 fingers motifs or less were included-yellow goes to methods). All multiple sequence alignments of the zinc finger regions of the C2H2-ZNF genes were generated using the program MUSCLE [54]. To resolve the challenging issue of comparing sequences containing multiple copies of a repeated finger motif sequence, that vary in copy number from gene to gene and whose patterns of duplication may differ from one species to the other, we used the following procedure. As a first step, we performed a multiple alignment of all the zinc finger regions of the C2H2-ZNF genes of interest; we removed all the sequences that were not unambiguously alignable and generated a new alignment allowing to visualize sequence similarity between the finger motifs of the various genes. As a second step, we performed individual pair-wise alignments of all the genes used in the first step. Then, we conducted a multiple alignment of the individual finger motifs from all C2H2-ZNF genes, to then determine if the similar finger motifs from a given C2H2-ZNF gene group together. The final alignment was derived from the above three alignments combined and then edited to remove gaps using the program GBLOCKS [55]. Maximum Likelihood (ML) and Bayesian Inference (BI) methods were used to infer the phylogenetic trees and estimate the clade support. For ML analysis, the program RAxML (RAxML-VI-HPC Version 2.2.1) [56] employing the WAG model of amino acid substitution was used to reconstruct the best tree. Bootstrapping of 100 datasets was implemented. The posterior probabilities were determined by a Bayesian MCMC method implemented in the program Mr. Bayes v.3.1 [57] to test the robustness of the topology of the tree inferred through ML. One million generations were run and the trees were sampled after every 10 generations.

To determine an appropriate outgroup for our analysis, we searched the nr database to look for close homologs in non-mammals using TBLASTN (e-value cut off 1e-4). In addition to the Xfin sequence from *Xenopus laevis*, we obtained a set of zinc finger genes from Chicken *(Gallus gallus*, Assembly WASHUC2) specifically selected for each human C2H2-ZNF cluster based on an extensive similarity search. To select the chicken outgroup, a TBLASTN (e-value cutoff 1e-4) search was done against the chicken genome using each of the human C2H2-ZNF sequences derived from the selected cluster of interest as a query. The top 10 hits for each query sequence were all analysed using a CD-HIT analysis (Identity threshold = 100%, 95% and 90%) [58] to produce a final set of non-redundant representative chicken sequences, all used as a part of the outgroup.

### Sequence analysis to confirm the loss of domains

In the case where loss of a domain was suspected, we conducted an extensive sequence analysis to rule out the possibility that these domains had remained undetected either due to a frame-shift or inadequate exon-intron splicing of the gene and thus inappropriate translation, preventing recognition by PROSITE [51]. Firstly, for each particular C2H2-ZNF genes where loss of an N-terminal domain was suggested, we systematically collected the nucleotide sequence of the region ranging from the stop of translation of the previous gene to the start of translation of the next gene. We conducted a TBLASTN search of this region using the amino acid sequence of the domain of interest (present in the corresponding orthologs and the consensus of the domains selected from randomly selected sequences) as a query to confirm the absence of the domain in the C2H2-ZNF gene of interest. Secondly, we obtained the exon-intron structure of these genes using the Ensembl Genome Browser [53], in order to search for exonic or intronic sequences which may exhibit significant identity with the nucleotide sequence of the domain of interest. For this purpose, we conducted a BLAST analysis of the individual exon and intron sequences with the nucleotide sequence of the various domains that are present in the corresponding orthologs.

### Flowchart of the study

Figure 1 summarizes the flowchart of the procedure for the analysis of C2H2-ZNF genes and clusters in mammals.

## Authors' contributions

HDT, GB and MA jointly conceived the project. HDT collected, compiled and analyzed all the data. HDT and MA wrote the first draft of the manuscript together. All authors jointly rewrote, edited all sections, read and approved the final manuscript.

## Supplementary Material

Additional File 1 **Supplementary Table 1. Catalog of human C2H2-ZNF genes**. The table lists the comprehensive catalogue of the 718 C2H2-ZNF genes in the human genome (21 pages) Click here for file

Additional File 2 **Supplementary Table 2. Distribution of the C2H2-ZNF genes on each human chromosome**. The table lists a comprehensive summary of the distribution of all C2H2-ZNF genes found as singletons or in clusters on each human chromosome and classified with respect to the various C2H2-ZNF sub-families (1 page). Click here for file

Additional File 3 **Supplementary Table 3. Organization of the C2H2-ZNF genes within human clusters**. The table lists the number and order of C2H2-ZNF genes from the various subfamilies within the 81 human C2H2-ZNF clusters (2 pages). Click here for file

Additional File 4 **Supplementary Table 4. Catalogue of human C2H2-ZNF clusters and their syntenic counterparts in mammals**. The table lists the comprehensive catalogue of the C2H2-ZNF genes from the 81 human clusters and their syntenic counterparts from other mammalian genomes chimpanzee, mouse, rat and dog (27 pages). Click here for file

Additional File 5 **Supplementary Figure 2. Comparison of human C2H2-ZNF clusters with their syntenic counterparts in other mammals**. The figure shows the comparison of the number of C2H2-ZNF genes in the 40 human clusters containing at least 3 C2H2-ZNF genes and their syntenic counterparts in four other mammals. For each human C2H2-ZNF cluster named on the graph and described in Additional File 3, the first number indicates the chromosome number and the second is the number attributed to that cluster on the chromosome. C2H2-ZNF clusters with three to five **(A) **or at least 6 genes **(B**) in human and their syntenic counterparts in chimpanzee, mouse, rat and dog are shown. This figure provides evidence of C2H2-ZNF differential species-specific expansion and gene loss in rodents. Click here for file

Additional File 6 **Supplementary Figure 1. Distribution of intergenic distances between the identified C2H2-ZNF genes**. The figure shows the distribution of intergenic distances between 718 C2H2-ZNF genes in the human genome. The intergenic distances between the consecutive C2H2-ZNF genes on each chromosome were calculated for each C2H2-ZNF gene of the human genome. For the 718 C2H2-ZNF genes, the number of C2H2-ZNF genes found within the range of intergenic distances indicated on the x axis is plotted on the y axis. For example, there are 108 C2H2-ZNF genes within 10 to 20 Kb from a consecutive C2H2-ZNF gene. Click here for file
