# Supplementary figures and images for: Evolution of C2H2-zinc finger genes and subfamilies in mammals: Species-specific duplication and loss of clusters, genes and effector domains
Source: BMC Evol Biol. 2008 Jun 18;8:176. doi: 10.1186/1471-2148-8-176 (PMC2443715; doi:10.1186/1471-2148-8-176)

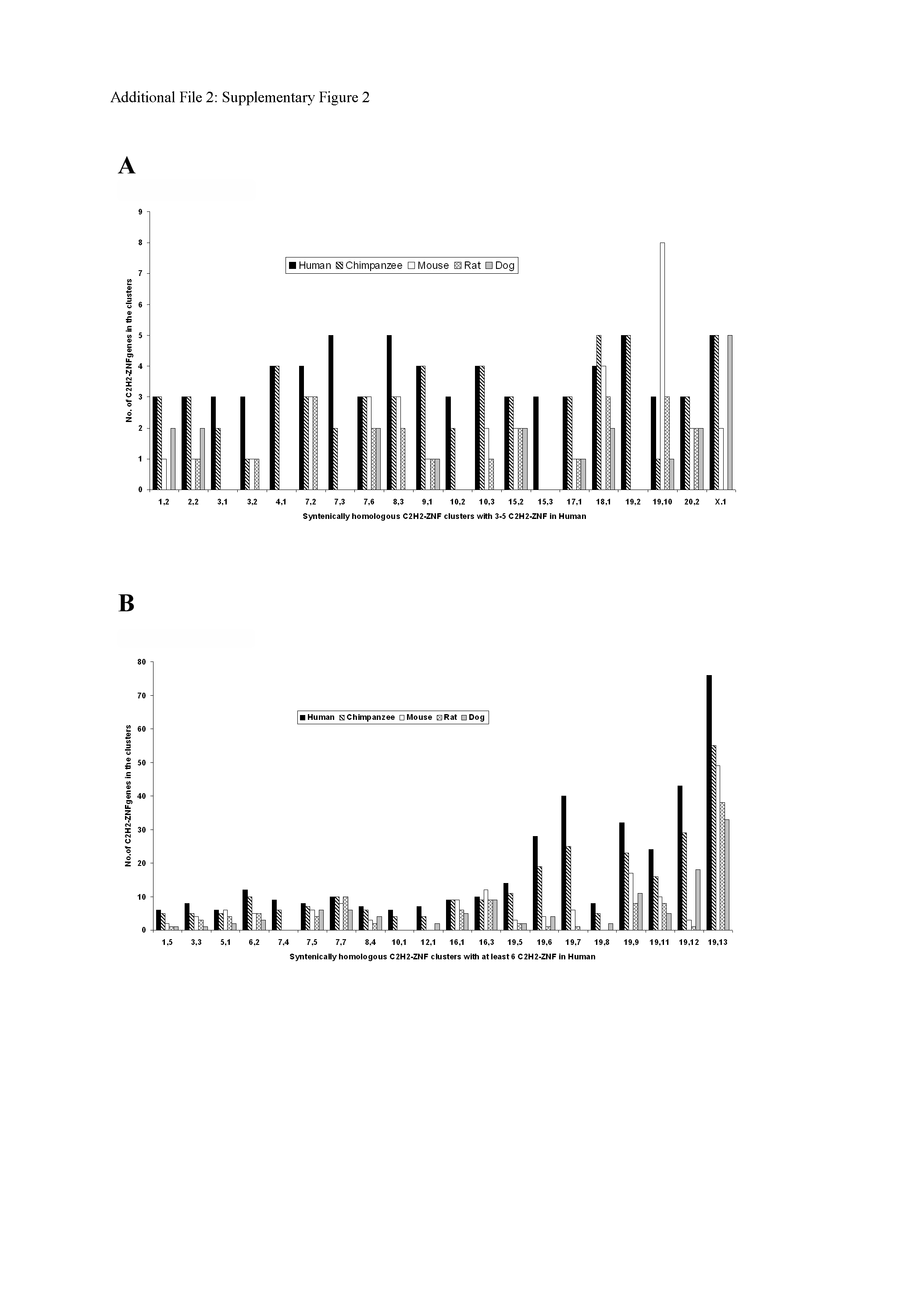

Supplement: Additional File 5 — Supplementary Figure 2. Comparison of human C2H2-ZNF clusters with their syntenic counterparts in other mammals. The figure shows the comparison of the number of C2H2-ZNF genes in the 40 human clusters containing at least 3 C2H2-ZNF genes and their syntenic counterparts in four other mammals. For each human C2H2-ZNF cluster named on the graph and described in Additional File 3, the first number indicates the chromosome number and the second is the number attributed to that cluster on the chromosome. C2H2-ZNF clusters with three to five (A) or at least 6 genes (B) in human and their syntenic counterparts in chimpanzee, mouse, rat and dog are shown. This figure provides evidence of C2H2-ZNF differential species-specific expansion and gene loss in rodents. [file 1471-2148-8-176-S5.png]

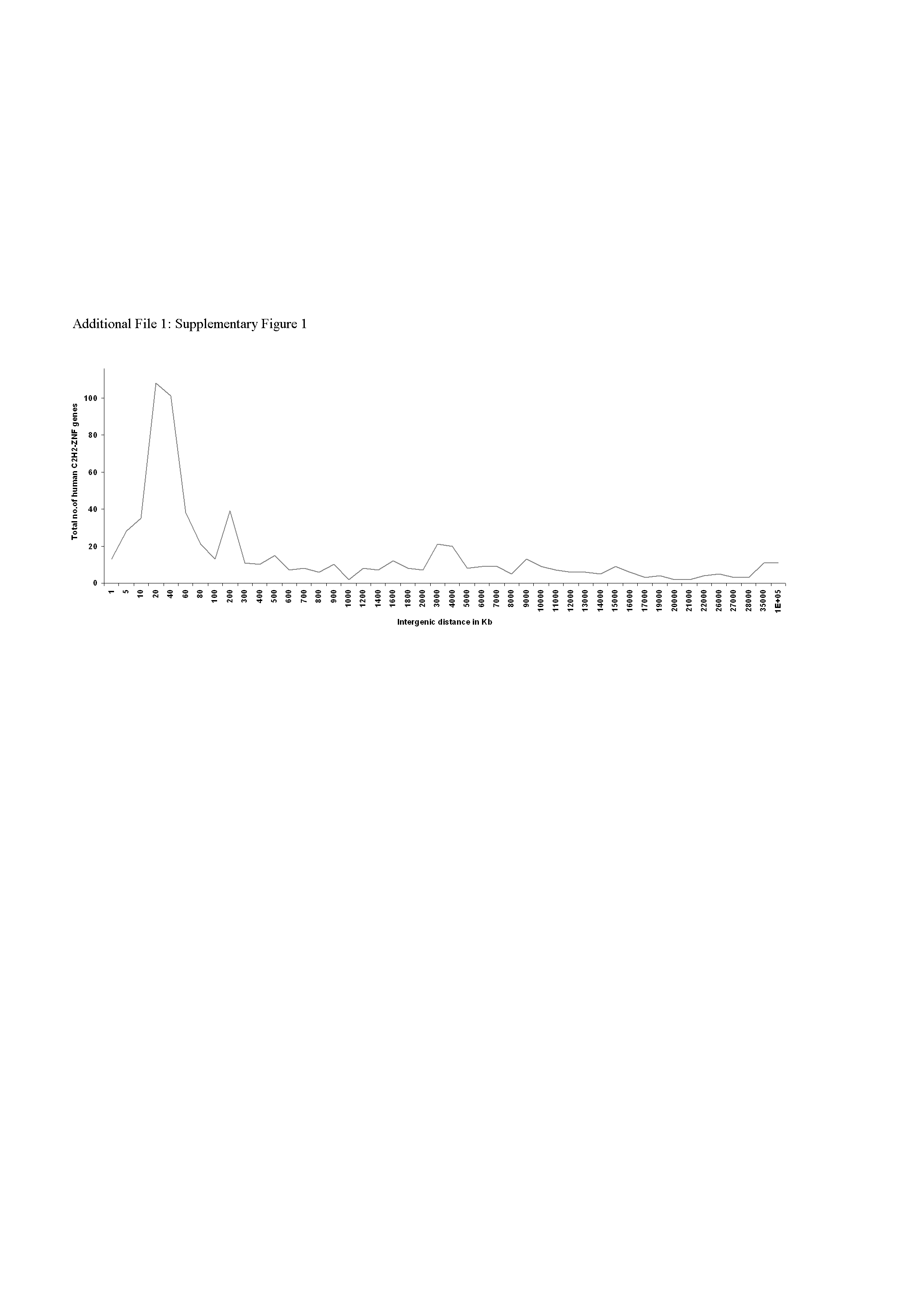

Supplement: Additional File 6 — Supplementary Figure 1. Distribution of intergenic distances between the identified C2H2-ZNF genes. The figure shows the distribution of intergenic distances between 718 C2H2-ZNF genes in the human genome. The intergenic distances between the consecutive C2H2-ZNF genes on each chromosome were calculated for each C2H2-ZNF gene of the human genome. For the 718 C2H2-ZNF genes, the number of C2H2-ZNF genes found within the range of intergenic distances indicated on the x axis is plotted on the y axis. For example, there are 108 C2H2-ZNF genes within 10 to 20 Kb from a consecutive C2H2-ZNF gene. [file 1471-2148-8-176-S6.png]
